# Use of information on disease diagnoses from databases for animal health economic, welfare and food safety purposes: strengths and limitations of recordings

**DOI:** 10.1186/1751-0147-53-S1-S7

**Published:** 2011-06-20

**Authors:** Hans Houe, Ian Andrew Gardner, Liza Rosenbaum Nielsen

**Affiliations:** 1 Faculty of Life Sciences, University of Copenhagen, Copenhagen, Denmark; 2 School of Veterinary Medicine, University of California, Davis, USA

## Abstract

Many animal health, welfare and food safety databases include data on clinical and test-based disease diagnoses. However, the circumstances and constraints for establishing the diagnoses vary considerably among databases. Therefore results based on different databases are difficult to compare and compilation of data in order to perform meta-analysis is almost impossible. Nevertheless, diagnostic information collected either routinely or in research projects is valuable in cross comparisons between databases, but there is a need for improved transparency and documentation of the data and the performance characteristics of tests used to establish diagnoses. The objective of this paper is to outline the circumstances and constraints for recording of disease diagnoses in different types of databases, and to discuss these in the context of disease diagnoses when using them for additional purposes, including research. Finally some limitations and recommendations for use of data and for recording of diagnostic information in the future are given. It is concluded that many research questions have such a specific objective that investigators need to collect their own data. However, there are also examples, where a minimal amount of extra information or continued validation could make sufficient improvement of secondary data to be used for other purposes. Regardless, researchers should always carefully evaluate the opportunities and constraints when they decide to use secondary data. If the data in the existing databases are not sufficiently valid, researchers may have to collect their own data, but improved recording of diagnostic data may improve the usefulness of secondary diagnostic data in the future.

## Introduction

Databases constructed directly for research purposes are often referred to as primary databases whereas databases originally constructed for other purposes are referred to as secondary databases [1]. Sometimes the distinction is not clear as data primarily collected for research are often combined with already existing data.

When disease data are collected directly for research purposes, there is often a very precise definition of the target conditions to be recorded and how to record them. However, the information on diseases in existing databases may have been collected for a number of different purposes and different practical and economical constraints and traditions that may limit their interpretation and usefulness when used as secondary data. Still, secondary databases can be very attractive as they can save time and resources spent on collecting new data. However, the circumstances and constraints should be clarified to assess whether the data fulfil the criteria when pursuing alternative uses of the data. In addition to general epidemiological criteria such as representativeness of the population, relevant time period etc., for diagnostic information there can often be a problem with the terminology or ‘ontology’ [2]. The ontology deals with questions concerning what entities exist or can be said to exist, and how such entities can be grouped, related within a hierarchy, and subdivided according to similarities and differences . Central to the ontology is the true status of the animals we want to identify (target condition) and how we interpret and translate diagnostic information into a practical case definition. Over time these issues have apparently not been addressed in a systematic way. Thus, diagnostic information can be related to clinical signs, pathology or the causative micro-organism. Further, the thoroughness of the diagnostic follow-up varies from just recording a single clinical sign to combining several observations and laboratory test results into a unified case definition that approximates the target condition of interest. The use of diagnoses and diagnostic tests has varied considerably over time. Also, codes for the same diagnosis may change over time or be categorised into more levels/groups or removed from the database so that a new case definition may have to be used, or, in the worst case, data are no longer sufficient to support the case definition.

Although, the usefulness of diagnostic information is usually described by accuracy measures such as sensitivity and specificity, this paper focuses on the importance of the ontology when we are using diagnostic information. The objective of this paper is to outline the circumstances and constraints for recording of disease diagnoses in different types of databases, and to discuss these in relation to the demands of the disease diagnoses when using them for additional purposes, including research. Further, we describe some limitations of secondary data and provide recommendations for use of data and for recording of diagnostic information in the future.

## Diagnoses and diagnostic tests

A diagnosis has been defined as “Identification of a disease or other specific health status of an individual or group of individuals showing clinical signs” [3]. The term is usually restricted to be under the interpretation of the clinician after all available information has been combined. Others have used the term independent of the diagnosticians’ role and training (clinician, pathologist or microbiologist). However, in these circumstances it may be more appropriate to talk about a diagnostic test. Thus, a diagnostic test in general terms has been described as ‘any device or process designed to detect, or quantify a sign, substance, tissue change, or body response in an animal’. Further, it is stated that “diagnostic tests are used to confirm or classify disease, guide treatment or aid in the prognosis of clinical disease” [4]. Thus, in its origin, the purpose of diagnoses and diagnostic tests has been rather narrow in aiming at treatment and making prognoses compared to the potential use in animal welfare, animal health economics and food safety.

Disease classification is essential in order to group patients and hence, to summarise experiences from patient groups rather than experiences from every individual patient. Ideally, a disease classification should be exhaustive and exclusive, but different perspectives over time have made it difficult to establish a logical system [5]. An overview of history with reference to human literature shows that from the 17th century, diseases were described as disease pictures and courses with emphasis on clinical signs and without knowledge of the underlying mechanisms. From 1800, patho-anatomical lesions for many diseases were characterised and the lesions were correlated with the clinical signs. Many disease designations used today refer to patho-anatomical lesions. In the last part of the 19th century, microbiologic agents were identified and hence diseases could be classified according to the causative infectious agents. In the last century, molecular biology methods have become increasingly important. For example, many diseases are characterised as genetic disorders, either as inherited or as mutations occurring later in life. Furthermore, in veterinary medicine, some diseases have been characterised according to the risk factors or circumstances, e.g. milk fever, shipping fever or loser cows, where the cows are unproductive compared to their herd mates. In some of these cases, the target condition is less precisely defined and multiple case definitions may be acceptable to describe or approximate the target condition.

It is not surprising that the increased information over time has affected the way we diagnose diseases as well as the terminology in use. However, because the different perspectives of characterising diseases using signs, lesions, causes and mechanisms all co-exist in the same disease classification or coding system, it can be difficult and sometimes impossible to interpret the data. For example, recordings of *Salmonella* infection rarely indicate whether the animal had diarrhoea or other clinical signs at time of testing. And with all the diagnostic test methods available today, we are often not sure whether a detected abnormality really means that the animal was diseased at the time of testing or recording.

## Criteria and constraints in Danish existing (secondary) databases as compared to primary databases

In Denmark, there are many veterinary related databases either owned by the Ministry of Food, Agriculture and Fisheries or owned by the agricultural sector [6]:

- National Central Husbandry Register (CHR)

- National medicine register (VetStat)

- National Veterinary practitioners register (VetReg)

- Control data register - from inspections in food and animals

- Laboratory tests register (national mandatory tests)

- Zoonosis register for *Salmonella* in swine (ZOOR)

- Poultry database (serology and ante mortem samples for *Salmonella*)

- Meat inspection database for cattle and swine, and BSE and TSE databases

- Cattle database (e.g. production control, mastitis control, disease registration, movement data)

- Swine production data

Many recordings of especially cattle diseases are based on treatment records from veterinary practitioners (supplemented by the farmer’s recordings). The motivation for the recordings is that the information is recorded anyway when veterinarians are preparing invoices for their services to the farmer, and it might provide an overview of which treatment-requiring disorders have been identified in the herd. Similarly, meat inspection data are readily obtained at the slaughterhouse, and one of the purposes has been to inform the farmer of the reason for a reduction in carcass price. If the purpose is to eradicate an infection, emphasis will be on the presence of the pathogen or immune response to the infection indicating recent or current infection. In a food safety program (e.g. Zoonosis register), focus will be on the presence or absence of the pathogen.

The above-mentioned circumstances and constraints in the secondary databases all have relevance to what is actually measured. For example, treatment records will favour recordings of evident clinical diseases and be hampered by treatment thresholds of the individual farmers and veterinarians. On the other hand, meat inspection data and data on occurrence of infectious agents may not necessarily indicate that the animal had a clinical problem.

Currently there is a desire to extend the use of existing databases for welfare characterisation and therefore existing databases are scrutinised for their suitability to provide welfare indicators. However, for the reasons mentioned above this should be done cautiously.

In contrast to the secondary databases, many databases are created as primary databases by researchers. For instance the cattle *Salmonella* database “CaSaDy”is essentially a collection of research datasets including repeated *Salmonella* laboratory results from all animals in 35 dairy herds in the so-called Kongeåproject that took place between 1999 and 2003 [7,8].

The research projects often have specific objectives of studying pathogenesis, risk factors, disease dynamics, production effects etc. Therefore they often use extended disease definitions, because dichotomous disease recordings may not suffice, and comprehensive characterisations may be needed. For example, Nielsen *et al.*[9] used the *Salmonella*-target conditions "carriers", "transiently infected" or "negative" (presumably non-infected) and based the practical case definitions on 4 repeated samples obtained over a period of at least 270 days. In another study, a total of 24 clinical parameters were given score values to evaluate the relationship between udder health and milk yield. The parameters were then analysed by factor analysis and related to milk production [10]. In order to elaborate a new disease entity, the so-called ‘loser cows’, Thomsen *et al.*[11] developed a clinical protocol for seven different clinical signs that were all given score values. Thus, many primary data have very complex target definitions. Furthermore, it has been shown that the accuracy of the tests for paratuberculosis varied substantially between the purposes of detecting an infected cow, an infectious cow or detection of a cow with production loss [12].

From these few examples, it is obvious that many research questions will have such a specific objective that investigators need to collect their own data. The question is then which questions and at what level of complexity can be answered using secondary data, and whether a small improvement in data quality can help in solving more questions.

## Limitations and recommendations for use of data and for recording of diagnostic information in the future

In Denmark, there has been a marked increase in dairy cow mortality [13]. However, it was not clear whether this increase was due to an increased number of ‘unassisted deaths’ or to an increased use of euthanasia. If it was due to an increased number of unassisted deaths, it could potentially be interpreted as an indication of poor welfare. If on the other hand, the increased mortality was due to an increase in the use of euthanasia it could be interpreted as an improvement of animal welfare, because fewer animals would have a long period with painful disease before dying unassisted. Therefore, the codes for death were extended in the cattle database to differentiate between unassisted death and euthanasia from January 1, 2008 [14]. However to evaluate the impact of changed mortality rates on animal welfare it must be recommended that all deaths be recorded together with the cause of death including information on duration and severity of clinical disease before the death/euthanasia.

Studies on the use of pre-collected register data show that register data can be used to classify herds according to welfare status. However, authors of the same studies stressed that the indicators have to be validated in field studies where the indicators are compared to more comprehensive evaluation of welfare in the herds [15].

In the Nordic countries, several validation studies have been performed on the national cattle databases. The sensitivity of using veterinary treatments as a measure of what can actually be seen in the herds by the farmer may vary considerably from one diagnosis to another [16]. However, if the validity remains constant over time for each diagnosis, such validation studies can be most useful for the future use of secondary databases. Thus, it would be beneficial if all the pre-collected data could be used more directly without the need for the time consuming and expensive validations on every occasion.

When designing a database it is important to consider how the data can be preserved for future use of data in unanticipated ways [2]. From the above-mentioned examples, it seems that often, just a little extra information or continued validation will yield a considerable improvement in the quality and therefore the potential use of secondary data. Regardless, researchers should always carefully evaluate the opportunities and constraints when they decide to use secondary data instead of measuring and collecting the data themselves.

In the ideal world, one could imagine a benefit if diseases (or conditions) were recorded together with a severity score for welfare (e.g. indicating level of pain and other welfare implications), a severity score for production loss etc. For example a paratuberculosis case could be recorded with low welfare implication but high production loss implication. However, it is still unclear whether the benefits of elaborating such a system will outweigh the effort and costs. Another problem with extended requirements to disease recordings is that this may lead to more errors and missing data due to lack of compliance from veterinarians and farmers. A way to improve compliance is to assure that the recordings can be used directly and easily as decision support for the farmer.

In human medicine, a systematised nomenclature of medical-clinical terms has been developed in the so-called ‘SNOMED CT’ as a set of standards to be used in hospitals [2]. However, in veterinary medicine there is a much broader scope than dealing with patients, because data are also used for evaluation of economic performance and welfare, and assessment of food safety. Therefore such a solution is not sufficient to serve the different demands to a database.

In order to answer the important question whether the benefits of elaborating more detailed data will outweigh the effort and costs, it seems logic to start with a thorough stakeholder analysis mapping the different needs, attitudes and visions. Hartig *et al*. [17] proposed such a stakeholder analysis to establish a health and disease database for the Danish horse population. Hopefully the future can such that such an approach can establish databases that are more functional and sustainable.

## Conclusion

Many research questions have a well-defined and specific objective and therefore investigators need to collect their own data to ensure that the questions are adequately addressed. However, there are also examples, where just a little extra information or continued validation could result in sufficient improvement of secondary data to be used for other purposes and thereby save time and resources otherwise spent on collecting new data. In any case, researchers should always carefully evaluate the opportunities and constraints when they decide to use secondary data instead of measuring and collecting the data themselves.

## Competing interests

The authors declare that they have no competing interests.
